# Sex-Specific trajectories of arterial stiffness: a large-scale description of 38 million real-world pulse wave velocity measurements

**DOI:** 10.3389/fcvm.2026.1808900

**Published:** 2026-05-28

**Authors:** Benjamin Vittrant, Pierre Boutouyrie, Rosa Maria Bruno

**Affiliations:** 1 Data Science, Withings, Issy-les-Moulineaux, France; 2 Inserm PARCC, Université Paris Cité, Paris, France; 3 Pharmacology Department, Hôpital Européen Georges Pompidou, DMU CARTE, Assistance Publique Hôpitaux de Paris, Paris, France

**Keywords:** MeSH vascular stiffness, pulse wave analysis, sex factors, reference values, telemedicine, child, aging

## Abstract

**Introduction:**

Arterial stiffness, measured by Pulse Wave Velocity (PWV), is a critical marker of cardiovascular risk. However, current reference values are often derived from limited clinical cohorts. This study aims to establish large-scale normative trajectories for PWV using real-world data.

**Methods:**

We analyzed anonymized data from 38,648,329 measurements collected via Withings smart scales in 1 196 712 unique users (681719 men and 514993 women) between 2023 and 2024, across Japan, North America, and Europe. PWV was estimated using ballistocardiography coupled with impedancemetry. We applied multivariate quantile regression to assess the impact of age, sex, and BMI on PWV.

**Results:**

There was a significant interaction between age and sex (*p* *<* *0.001*). Men exhibited higher PWV values in early adulthood after adolescence compared to women. However, a distinct crossover was observed around the fifth decade of life, after which women displayed a steeper rate of arterial stiffening, eventually surpassing men. Age, sex and BMI were independently associated with PWV.

**Discussion:**

This observational study provides the largest real-world dataset on PWV to date. The results illustrate in an observational way the sex dependent “vascular aging crossover” likely associated with hormonal change at adolescence and later menopause. This finding demonstrates the utility of connected devices in large-scale cardiovascular epidemiology.

## Introduction

Arterial stiffness (AS), quantified by pulse wave velocity (PWV), has been recognized for decades as a key predictor of cardiovascular risk (1–5). Vascular aging, arguably the most meaningful clinical descriptor derived from PWV, has been the subject of dense literature exploring its association with cardiovascular events, hypertension, and diabetes (6–8).

As with any medical parameter, it is crucial to differentiate between technical and biological variability. Furthermore, within biological variability, one must distinguish pathological deviations from the normal physiological range. To address this, various teams have established technical benchmarks and published normative data, ranging from large epidemiological datasets (9–11) to more targeted, age-related values (12). These normative values, when specific to a measurement method and population, are needed since they provide a baseline, thus enabling the comparison of an individual's data and its further use as a clinical tool for stratifying risk and guiding intervention (13).

One important limitation for the application of those concepts in the clinic is the inadequacy of lab measurements (gold standard PWV) in clinical practice, not to say at population level. The discrepancy between data gathered in controlled clinical settings and “real-world data” (RWD) constitutes a real gap, not limited to therapeutic interventions, since reported efficacy in trials often fails to translate to equivalent real-world effectiveness (14, 15). This discrepancy also applies to diagnostic and prognostic parameters. Data from controlled studies, often derived from small or highly-selected cohorts, may not fully capture the spectrum of values seen in the “free range” general population (e.g., typically sex dose efficiency), thereby limiting generalizability.

Both controlled-study and real-world approaches are necessary and complementary. With this in mind, In the present study, we aimed at providing evidence at population level using connected health devices that allows for the collection of high-frequency, longitudinal data in a home setting (“real-world evidence”). This study leverages a massive dataset of over 38 million measurements from smart bathroom scales to: (1) describe normative PWV values across diverse geographies, and (2) characterize the distinct trajectories of vascular aging in men and women.

## Materials and methods

### Measuring devices

The PWV data come from all our scale models with PWV features available. The technology was compared initially (16) with the Sphygmocor device and the conclusion was that it estimated the carotid-femoral PWV with acceptable accuracy and precision. At our knowledge we found one paper controlling the PWV measurement according to the standards of ARTERY (https://www.arterysociety.org/) society (17) acknowledging its usability. Unlike traditional tonometry, the smart scale measures PWV using a combination of Ballistocardiography (BCG) and Impedance Plethysmography (IPG). All Withings's devices are CE certified and medical features are subject to validation clinical trials according to medical legislation. More details are available at https://www.withings.com/eu/en/compliance.

### Dataset

We analyzed a retrospective dataset of 38,648,329 PWV measurements collected from 1 196 712 unique users (681719 men and 514993 women) between 2023 and 2024. The study population spanned four major geographic regions: North America (USA, Canada, Mexico), Western Europe (France, Germany, UK), Southern Europe (Italy, Spain, Portugal, Greece), and Asia (Japan). Stringent quality control measures were applied to the raw dataset. Notably, measurements from users aged <7 years were excluded. Exploratory analysis revealed a median weight of ∼50 kg in this age group, which is biologically implausible for infants or young children. This problem can be observed in Supplementary file 1 where we plotted the weight per age to illustrate the problem. This discrepancy likely results from parents holding their children while standing on the scale, thereby confounding the measurement. Consequently, these data points were removed to preserve the integrity of the analysis. Additionally bmi data were filtered to remove aberrant values over 60 and inferior to 8 kg/m^2^). No filters were applied to PWV.

### Tools

The data exploration was performed using R v4.3.2 (18, 19), Visual Code Studio (VCS) v1.106.0 (20), rstudio build 494 (21), and Quarto v1.2 (https://quarto.org/).

### Visualization and statistical analysis

**Figure 1:**
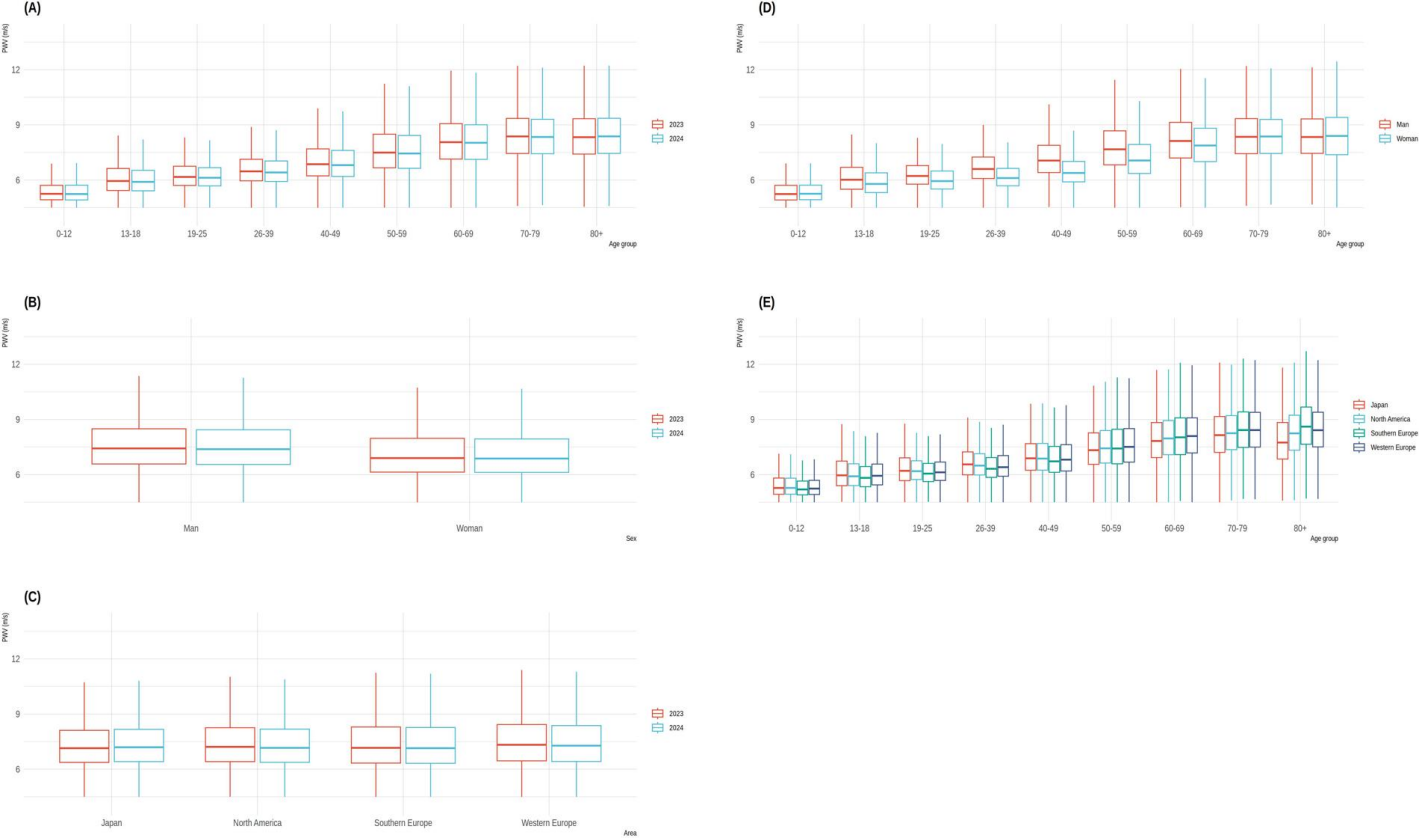
Exploratory analysis of pulse wave velocity (PWV) distribution across demographic and geographic variables. This multi-panel figure illustrates the distribution of PWV (m/s) categorized by temporal, biological, and regional factors. Boxplots represent the median (horizontal center line), interquartile range (box), and 95% confidence intervals or whiskers (vertical lines). Panel **(A)** compares PWV by age group and year (2023 in red; 2024 in blue), demonstrating a progressive increase in arterial stiffness across the lifespan. Panel **(B)** presents sex-specific distributions, while panel **(C)** displays regional variations across Japan, North America, Southern Europe, and Western Europe for both study years. Panels **(D)** and **(E)** further delineate these trends by illustrating the interaction between age and sex, and age and geographic region, respectively, highlighting the acceleration of arterial aging in specific cohorts.

**Figure 2:**
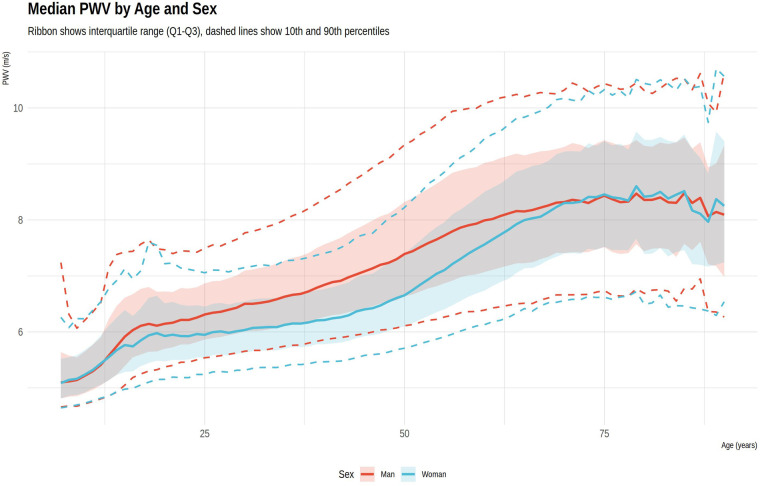
Age-Related trajectories of median pulse wave velocity (PWV) stratified by Sex. This figure illustrates the longitudinal progression of arterial stiffness across the lifespan, with data bifurcated by biological sex (Men in red; Women in blue). The solid lines represent the median PWV (m/s), while the shaded ribbons indicate the interquartile range (IQR, Q1–Q3), and the dashed lines denote the 10th and 90th percentiles.

**Figure 3:**
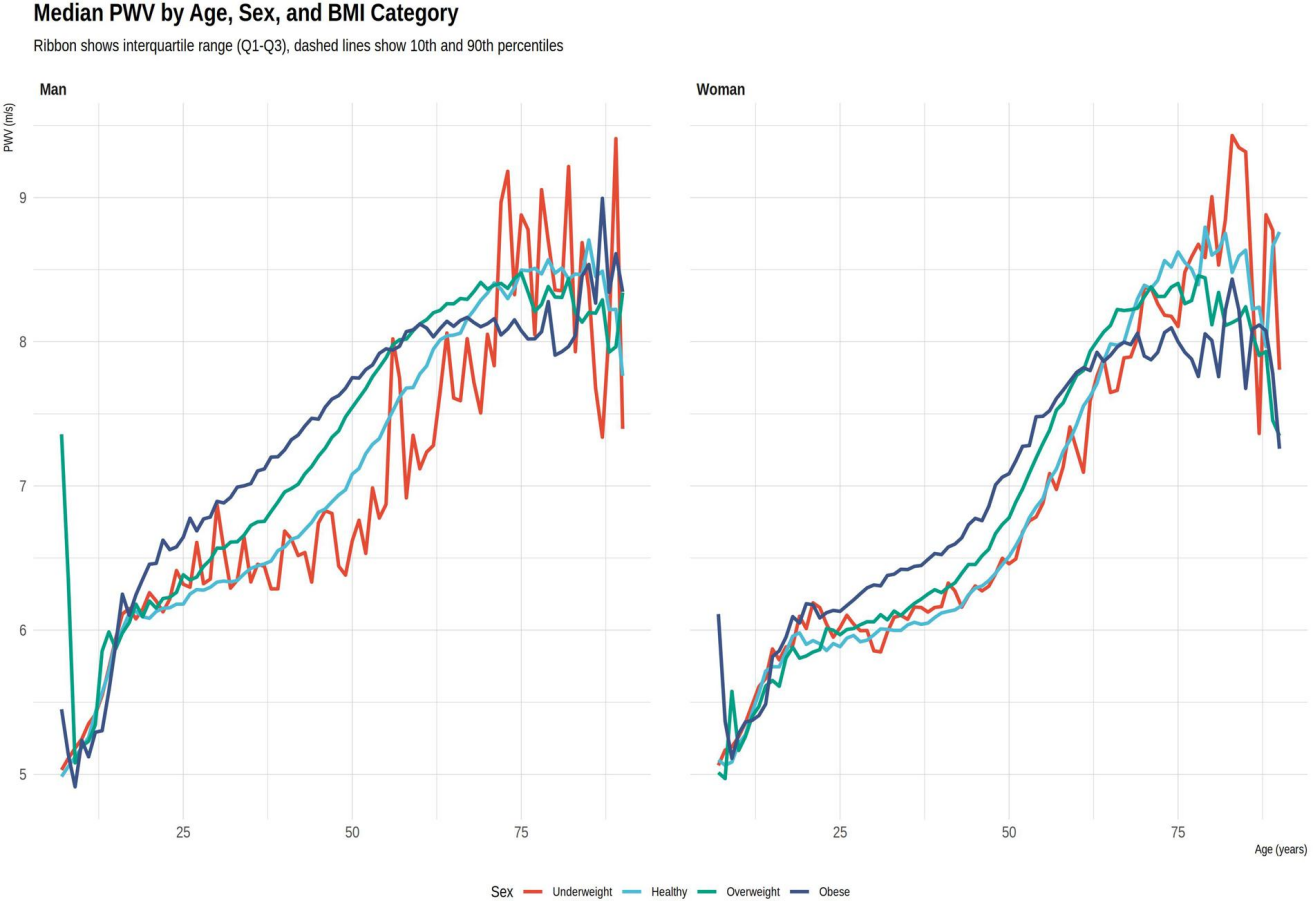
Interaction of Age, Sex, and body mass Index (BMI) on pulse wave velocity (PWV). This dual-panel plot evaluates the impact of BMI categories (Underweight, Healthy, Overweight, and Obese) on the age-related increase of PWV (m/s) for men (left) and women (right). Median values for each BMI category are plotted across the age spectrum. In both sexes, an “obesity gradient” is visible in mid-life, with the obese cohort (dark blue line) generally maintaining higher PWV values compared to the healthy-weight cohort (light blue line). The underweight cohort (orange line) exhibits significant fluctuations, particularly in the later stages of life, likely due to the smaller sample size.

Descriptive boxplot were generated for demographic variables (Age, Weight, BMI, year) and are available in Figure 1. Visual trends were assessed using smoothed curves to observe non-linear aging trajectories (Figures 2, 3).

Data inspection revealed a significant correlation (pearson > 0.6) between geographical area and weight. This aligns with established epidemiological data, where obesity prevalence is highest in North America, followed by Western Europe, Southern Europe, and Japan. Plus our dataset lacks granular socio-economic variables (e.g., healthcare access, diet quality) to adjust for these regional disparities. Consequently we choose to omit geographical data in order to focus on biological determinants within modelization. BMI was selected over weight for this analysis because it better reflects weight-related health risks, despite its imperfections (22). The strong correlation between these two features also made their simultaneous inclusion in the linear model inappropriate.

To capture the complex physiological dynamics observed in the exploratory analysis (Figures 2, 3), we employed quantile regression (QR) to model the conditional distribution of Pulse Wave Velocity (PWV). This was also indicated because preliminary check showed non normality for PWV and problems with residuals in initial linear modeling. Unlike Ordinary Least Squares (OLS), which estimates the conditional mean, QR allows for the examination of predictors across the entire spectrum of arterial stiffness. We specified a non-linear model:

PWV∼poly(Age,2)*Sex+BMI

This formulation allowed us to model the sex-specific “S-curve” through a second-order polynomial interaction, while treating BMI as a linear additive covariate. Within the model all data were pulled. We didn't consider any individual effect since with the high dimensionality this effect has less influence on the overall results.

### Ethical consideration & anonymisation

Data were anonymized following the GDPR guidelines (27) (https://www.zostero.org/google-docs/?zvUSYD) edited by the French data privacy control institution (https://www.zotero.org/google-docs/?pdlvV3) (26). Randomness was added to PWV, weight and age (0.1 between 0 and 1) and then data were rounded to units thus to follow the principle of randomization/generalization. Only aggregate results derived from these steps were used.

### Generative AI

Text and code improvements were made using Gemini 2.5 pro and Copilot integrated within Visual Code Studio (VCS).

## Results

### Demographics

The demographic characteristics of the cohort were as follows. The geographic distribution was uneven, with the majority of data originating from Western Europe (*N* ∼21.8 million) and North America (*N*∼12.2 million), while Japan (*N*∼2.3 million) and Southern Europe (*N*∼2.2 million) represented smaller sub-cohorts (see Table 1). The Sex distribution showed the cohort was predominantly male, accounting for 68% of the measurements. The overall mean Body Mass Index (BMI) was 26.0 ± 4.6 kg/m^2^ (Table 2). However, significant regional heterogeneity was observed, with the Japanese cohort being the leanest (Mean BMI: 23.5 kg/m^2^) and the North American cohort having the highest average mass (Mean BMI: 26.9 kg/m^2^). The distribution across standard BMI Categories for the overall dataset was: Underweight (1.5%), Healthy Weight (44%), Overweight (38%), and Obese (17%).

**Table 1:** The table presents the mean (standard deviation) for continuous characteristics (PWV, weight, Fat mass, BMI, Age) and the count (percentage) for categorical variables (BMI group and Sex) across geographical areas.

Characteristic		Overall *N* = 38,531,985	Japan *N* = 2,348,548	North America *N* = 12,136,894	Southern Europe *N* = 2,263,605	Western Europe *N* = 21,782,938
PWV (m/s)		7.5 (1.4)	7.4 (1.4)	7.4 (1.4)	7.4 (1.5)	7.5 (1.5)
Weight (Kg)		79.4 (16.9)	66.8 (13.0)	81.7 (17.5)	77.4 (15.3)	79.7 (16.4)
Fatmass (Kg)		19.9 (9.7)	15.2 (6.8)	21.2 (10.4)	18.0 (8.7)	19.9 (9.4)
Fatmass (%)		24.5 (8.9)	22.3 (7.3)	25.4 (9.2)	22.8 (8.7)	24.4 (8.8)
Body mass index		26.1 (4.4)	23.5 (3.6)	26.9 (4.7)	25.5 (4.0)	26.0 (4.3)
Body mass index (Group)						
	Underweight	584,699 (1.5%)	109,859 (4.7%)	133,676 (1.1%)	36,529 (1.6%)	304,635 (1.4%)
	Healthy	16,976,464 (44%)	1,563,070 (67%)	4,523,347 (37%)	1,095,835 (48%)	9,794,212 (45%)
	Overweight	14,532,149 (38%)	546,815 (23%)	4,840,796 (40%)	856,713 (38%)	8,287,825 (38%)
	Obese	6,438,673 (17%)	128,804 (5.5%)	2,639,075 (22%)	274,528 (12%)	3,396,266 (16%)
Age (years)		51.8 (12.9)	51.2 (11.6)	50.8 (13.0)	51.7 (12.4)	52.4 (13.1)
Sex						
	Man	26,301,898 (68%)	1,709,204 (73%)	8,220,208 (68%)	1,661,792 (73%)	14,710,694 (68%)
	Woman	12,230,087 (32%)	639,344 (27%)	3,916,686 (32%)	601,813 (27%)	7,072,244 (32%)

**Table 2:** The table presents the mean (standard deviation) for continuous characteristics (PWV, weight, Fat mass, Age) and the count (percentage) for categorical variables (areas and Sex) across BMI groups.

Characteristic		Overall *N* = 38,531,985	Underweight *N* = 584,699	Healthy *N* = 16,976,464	Overweight *N* = 14,532,149	Obese *N* = 6,438,673
PWV (m/s)		7.5 (1.4)	6.9 (1.3)	7.2 (1.4)	7.7 (1.5)	7.8 (1.4)
Weight (Kg)		79.4 (16.9)	48.8 (7.3)	67.9 (9.8)	83.8 (9.8)	102.7 (14.4)
Fatmass (Kg)		19.9 (9.7)	7.1 (3.5)	13.7 (4.9)	21.2 (5.9)	34.4 (9.5)
Fatmass (%)		24.5 (8.9)	14.6 (6.8)	20.5 (7.4)	25.6 (7.4)	33.6 (8.0)
Body mass index		26.1 (4.4)	17.5 (1.1)	22.6 (1.6)	27.1 (1.4)	33.5 (3.2)
Age (years)		51.8 (12.9)	46.7 (17.9)	51.2 (13.4)	52.6 (12.5)	52.1 (11.9)
Sex						
	Man	26,301,898 (68%)	155,214 (27%)	10,293,936 (61%)	11,211,678 (77%)	4,641,070 (72%)
	Woman	12,230,087 (32%)	429,485 (73%)	6,682,528 (39%)	3,320,471 (23%)	1,797,603 (28%)
Area						
Japan		2,348,548 (6.1%)	109,859 (19%)	1,563,070 (9.2%)	546,815 (3.8%)	128,804 (2.0%)
	North America	12,136,894 (31%)	133,676 (23%)	4,523,347 (27%)	4,840,796 (33%)	2,639,075 (41%)
	Southern Europe	2,263,605 (5.9%)	36,529 (6.2%)	1,095,835 (6.5%)	856,713 (5.9%)	274,528 (4.3%)
	Western Europe	21,782,938 (57%)	304,635 (52%)	9,794,212 (58%)	8,287,825 (57%)	3,396,266 (53%)

### Exploratory analysis of PWV distributions

The exploratory analysis of Pulse Wave Velocity (PWV) reveals a consistent, age-dependent increase in arterial stiffness across all studied cohorts (Figure 1). Initial comparisons between data collection years 2023 and 2024 show stable distribution patterns, suggesting high reproducibility of the measurements over the two-year period (Figures 1A–C). When stratified by sex, men consistently exhibit higher median PWV values compared to women across the total population (Figure 1B). Regional analysis indicates relative homogeneity in PWV distributions across Japan, North America, and Europe, though minor variations in the rate of age-related stiffening appear when interacting age with geographic location (Figure 1E).

A more granular examination of age-related trajectories confirms a non-linear progression of arterial stiffness (Figure 2). In early-to-mid adulthood (ages 20–50), men maintain a higher median PWV than women, a gap supported by the distinct separation of the interquartile ribbons. However, this sex difference significantly diminishes in late adulthood. Starting approximately at age 65, the rate of PWV increase with age in women accelerates, leading to a convergence of median values by age 75. The widening of the 10th and 90th percentile bands in the elderly cohort (80 + years) highlights increased inter-individual variability in vascular aging during senescence.

The interaction between Body Mass Index (BMI) and age further modulates the PWV profile (Figure 3). In both men and women, a clear “metabolic gradient” is observed, particularly between the ages of 30 and 70, where individuals in the obese category (BMI > 30) exhibit higher median PWV values compared to their healthy-weight counterparts. This increased arterial stiffness suggests that excess adiposity acts as a significant accelerator of vascular aging. While the overweight and obese cohorts show relatively smooth trajectories due to higher sample density, the underweight cohort (orange line) exhibits marked volatility across the age spectrum. This fluctuation is attributed to a significantly lower sample size (*N*) within this BMI category, making the median PWV highly sensitive to individual variance and measurement noise rather than a specific biological trend. Similarly, the convergence and increased instability of all BMI curves beyond age 75 likely reflect the combined effects of reduced data density in the oldest-old population and potential survivor bias.

### Quantile regression analysis of PWV determinants

**Figure 4:**
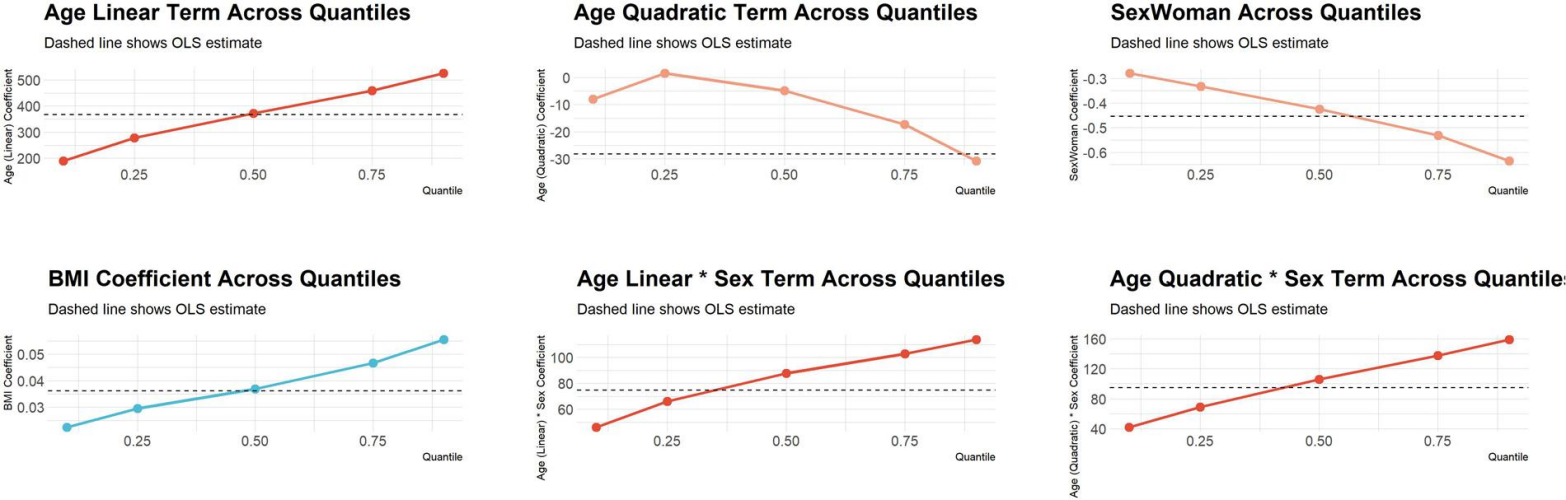
Quantile regression coefficients for pulse wave velocity (PWV) across the distribution. This figure illustrates the variation in regression coefficients for each model predictor as a function of the quantile (τ) from 0.10 to 0.90. The horizontal dashed lines represent the corresponding Ordinary Least Squares (OLS) estimate, while the solid lines with markers display the quantile-specific coefficients for the linear component of age (top left), the quadratic component of age (top middle), biological sex (top right), Body Mass Index (bottom left), and the interaction terms between biological sex and the linear (bottom middle) and quadratic (bottom right) components of age. Each panel demonstrates how the magnitude and direction of each variable's influence shifts across the different percentiles of the PWV spectrum.

Age was modeled using a second-degree polynomial to account for the non-linear acceleration of arterial stiffening. Results are presented in Table 3 with coefficient values for each term and illustrated in Figure 4.

**Table 3:** Quantile regression coefficient table associated with our presented model.

Characteristic	Q10	95% CI	*p*-value	Q25	95% CI	*p*-value	Q50	95% CI	*p*-value	Q75	95% CI	*p*-value	Q90	95% CI	*p*-value
Age (years)															
* Age (years)*	190	187, 194	<0.001	278	274, 281	<0.001	373	369, 376	<0.001	460	455, 464	<0.001	526	519, 532	<0.001
* Age (years)^2^*	−8.0	−11, −5.2	<0.001	1.6	−1.1, 4.3	0.2	−4.8	−7.7, −2.0	<0.001	−17	−21, −13	<0.001	−31	−37, −25	<0.001
Sex															
* Man*	-	-	-	-	-	-	-	-	-	-	-	-	-	-	-
* Woman*	−0.28	−0.29, −0.27	<0.001	−0.33	−0.34, −0.32	<0.001	−0.42	−0.43, −0.42	<0.001	−0.53	−0.54, −0.52	<0.001	−0.63	−0.65, −0.62	<0.001
Body Mass Index	0.02	0.02, 0.02	<0.001	0.03	0.03, 0.03	<0.001	0.04	0.04, 0.04	<0.001	0.05	0.05, 0.05	<0.001	0.06	0.05, 0.06	<0.001
Age (years) * Sex															
Age (years) * Woman	46	40, 53	<0.001	66	60, 72	<0.001	88	82, 94	<0.001	103	95, 111	<0.001	114	103, 125	<0.001
Age (years)^2^ * Woman	42	37, 47	<0.001	69	64, 74	<0.001	106	101, 111	<0.001	137	130, 145	<0.001	159	149, 169	<0.001

The linear component of the age effect was a significant predictor across all segments of the distribution (*p* < 0.001), with the magnitude of the coefficient increasing substantially at higher quantiles. Specifically, the age-related effect rose from 190 at the 10th percentile to 526 at the 90th percentile. This widening gap indicates that age has a disproportionately larger impact on individuals who already possess higher baseline levels of arterial stiffness. The quadratic term was also significant for most quantiles (*p* < 0.001), supporting the use of a non-linear model to represent the vascular aging trajectory (Figure 4).

Declared biological sex significantly influenced PWV across the entire spectrum (*p* < 0.001). Being female was associated with a lower PWV compared to being male, an effect that became more pronounced in the upper quantiles (shifting from −0.28 at Q10 to −0.63 at Q90). Furthermore, significant interaction terms between the polynomial age components and sex (poly(Age, 2) * Woman) were observed (*p* < 0.001). These interactions indicate that the shape and rate of age-related vascular changes are sex specific, confirming that the stiffening process follows different trajectories for men and women as they age.

BMI exhibited a consistent positive influence on PWV across all quantiles (*p* < 0.001). Similar to the age effect, the association of BMI was amplified in the higher percentiles of the distribution, with coefficients rising from 0.02 at Q10 to 0.06 at Q90. This finding suggests that while elevated BMI is a universal risk factor for increased PWV, its contribution to arterial stiffening is most severe in the “stiffest” segment of the population.

We also provide some results about asymptotic behaviors for quantile regression. At first glance, in order to evaluate the time it would take to run the QR we took 1/10^4^ of the rows, then 1/10^3^ then 1/10^2^ for all the data. We observed that from 1/10^2^ results were mostly identical when adding more data. There were no numerical differences in coefficients from 1/10 to 1/1 data selected. We provided Supplementary files to illustrate this observation with 1/10 of the data in Supplementary file 2, 1/100 of the data in Supplementary file 3, 1/1000 of the data in Supplementary file 4 and 1/10000 of the data in Supplementary file 5.

## Discussion

This study represents the largest epidemiological analysis of arterial stiffness to date, utilizing over 38 million real-world measurements to characterize vascular aging trajectories. By employing quantile regression, we moved beyond population averages to reveal that the primary drivers of PWV—age, sex, and BMI—act with varying intensity depending on an individual's position in the physiological distribution. This approach confirms that traditional OLS models consistently underestimate risk factors for the most vulnerable individuals in the upper quantiles of the population. It is also well known that the risk factors in medicine are rarely really linear but more with amplificatory effects in general.

The most notable insight is the precise characterization of the possible sexual difference in vascular aging. This is in contrast with the current evidence from the Reference value project, where women had similar PWV values than men. This holds true in a more recent epidemiological survey in children and young adults (23), using reference methods. However, a recent population survey in Austria reported a higher incidence of EVA in women (24). In the latter, mean age was 52, close to the age at which the slope changes between sexes in our study. Both reference value projects were based mainly on cohorts constituted at hospitals, which contrast with general population surveys (as in Austria), or customer databases such as our study.

The data corroborates the “protective” female phenotype during middle age, but QR reveals that this protection is most significant for individuals in the higher risk quantiles, where the sex gap is widest and persists longest. The critical crossover observed around the fifth decade coincides with the typical age of menopause and the loss of hormonal protection, leading to an accelerated stiffening rate in women. We hypothesize that this divergence, which begins as early as adolescence, could provide a robust benchmark for identifying “Early Vascular Aging” (EVA) decades before clinical manifestations of cardiovascular disease. Note that this hypothesis originated during the curation and validation of a large-scale, multi-center aggregate dataset intended for public release (25). Preliminary analysis of this open-access resource revealed a distinct sex-based divergence in pulse velocity beginning in adolescence; consequently, this study was designed to formally investigate these observations at individual level as a potential benchmark for Early Vascular Aging (EVA).

Furthermore, the significant slope heterogeneity observed for BMI carries substantial clinical implications. Because BMI is a much stronger predictor of PWV at the 90th percentile than at the 10th, weight management interventions may yield the highest cardiovascular benefits for individuals already exhibiting high arterial stiffness. The non-linear age dynamics also highlight a physiological “ceiling” effect, where the acceleration of stiffness eventually slows down, particularly in high-risk cohorts. While the study is limited by its observational nature and potential selection bias among connected device users, the scale of the dataset provides a high-fidelity view of real-world vascular aging. These findings underscore the utility of connected health devices as scalable tools for public health monitoring and early risk detection across the lifespan.

The study population consists of Withings connected device users, a cohort subject to the selection biases inherent in digital health consumerism. A significant constraint of this dataset is the lack of granular information regarding participant habitus, baseline clinical risk factors, and longitudinal medical histories. While health-oriented wearable users often exhibit a ‘healthy user bias'—characterized by proactive health-seeking behaviors—the population remains inherently heterogeneous. For example, the inclusion of individuals utilizing blood pressure monitors likely introduces a sub-cohort with elevated cardiovascular risk or pre-existing hypertension. Although this population typically skews toward higher sociodemographic strata, the relative affordability of Withings hardware compared to other international mHealth competitors may broaden the socioeconomic spectrum, partially mitigating the homogeneity common in wearable research. Nonetheless, as these devices are primarily self-procured rather than clinically mandated, the cohort likely reflects high levels of health literacy and intrinsic motivation. Consequently, the generalizability of these findings remains subject to these unobserved confounding variables, necessitating cautious interpretation.

## Conclusion

This study, the largest real-world analysis of Pulse Wave Velocity (PWV) to date (*N* > 38 million measurements in 1, 2 million individuals), establishes robust normative trajectories and highlights a profound sexual dimorphism in vascular aging. We observed a critical ‘vascular aging crossover' between 55 and 60 years of age, where the accelerated arterial stiffening rate in post-menopausal women, thus converging toward male values. Moreover, our data suggests the onset of this sexual divergence is evident around adolescence, providing an unprecedented benchmark for identifying ‘Early Vascular Aging' (EVA) in pediatric populations. This work shows connected health devices could be a scalable tool for public health monitoring and risk stratification across the lifespan, especially in places like the daily home where other tools cannot be used.

## Limitations

• **Observational**

As all retrospective observational studies, we are limited by the bias we have no control over and the lack of confounding medical factors at our disposal.

• **Measurement technique**

While validated, standing BCG measurement is sensitive to posture and differs slightly from supine cf-PWV.

• **Data categories**

Underweight category is underrepresented and cofounded with underage individuals. It should be analyzed carefully.

## Data Availability

The datasets presented in this article are not readily available because of ethical concerns. Requests to access the datasets should be directed to the corresponding author.

## Supplementary material

Supplementary File 1 Distribution of body weight by age. A linear representation of weight-for-age reveals significant outliers within the 0–3 year cohort, characterized by weight values exceeding 50 kg. These biological implausibilities likely stem from measurement error, specifically "proxy interference" where caregivers are weighed simultaneously with the infant.

Supplementary File 2 Quantile regression coefficients for pulse wave velocity (PWV) across the Distribution. This figure is the same plot as in Figure 4 except we used only 1/10 of the data.

Supplementary File 3 Quantile regression coefficients for pulse wave velocity (PWV) across the distribution. This figure is the same plot as in Figure 4 except we used only 1/100 of the data.

Supplementary File 4 Quantile regression coefficients for pulse wave velocity (PWV) across the distribution. This figure is the same plot as in Figure 4 except we used only 1/1000 of the data.

Supplementary File 5 Quantile Regression Coefficients for Pulse Wave Velocity (PWV) across the Distribution. This figure is the same plot as in Figure 4 except we used only 1/10000 of the data.
