# Supplementary figures and images for: Sex-Specific trajectories of arterial stiffness: a large-scale description of 38 million real-world pulse wave velocity measurements
Source: Front Cardiovasc Med. 2026 May 28;13:1808900. doi: 10.3389/fcvm.2026.1808900 (PMC13253226; doi:10.3389/fcvm.2026.1808900)

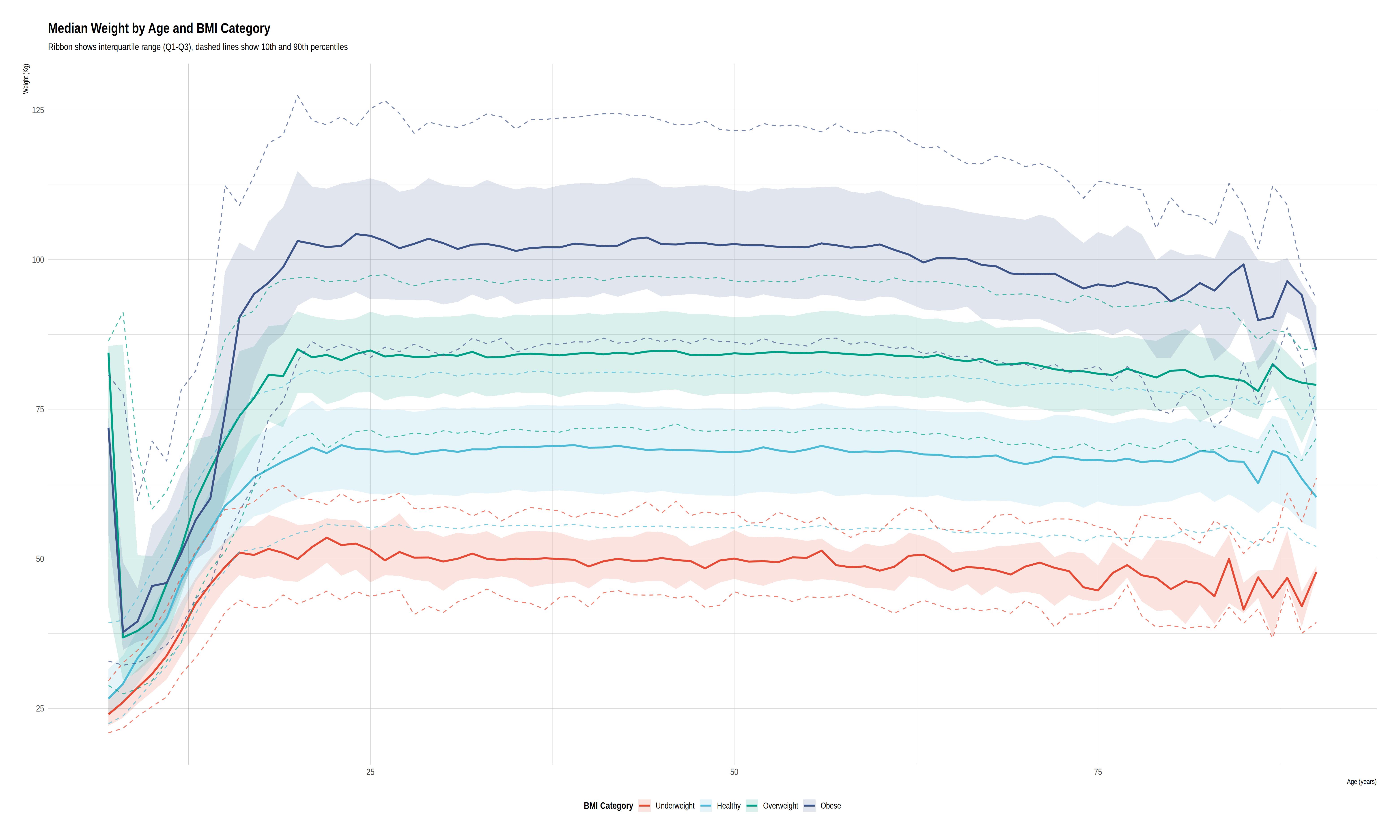

Supplement: Supplementary File 1 — Distribution of body weight by age. A linear representation of weight-for-age reveals significant outliers within the 0–3 year cohort, characterized by weight values exceeding 50 kg. These biological implausibilities likely stem from measurement error, specifically "proxy interference" where caregivers are weighed simultaneously with the infant. [file Image1.jpeg]

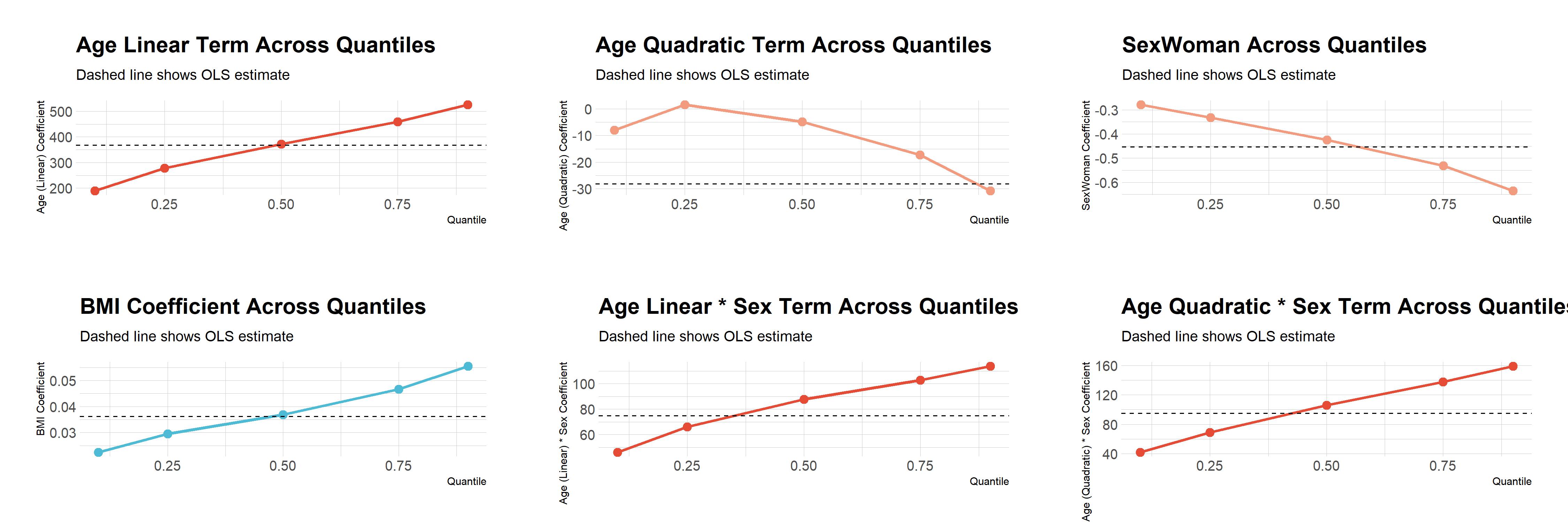

Supplement: Supplementary File 2 — Quantile regression coefficients for pulse wave velocity (PWV) across the Distribution. This figure is the same plot as in Figure 4 except we used only 1/10 of the data. [file Image2.jpeg]

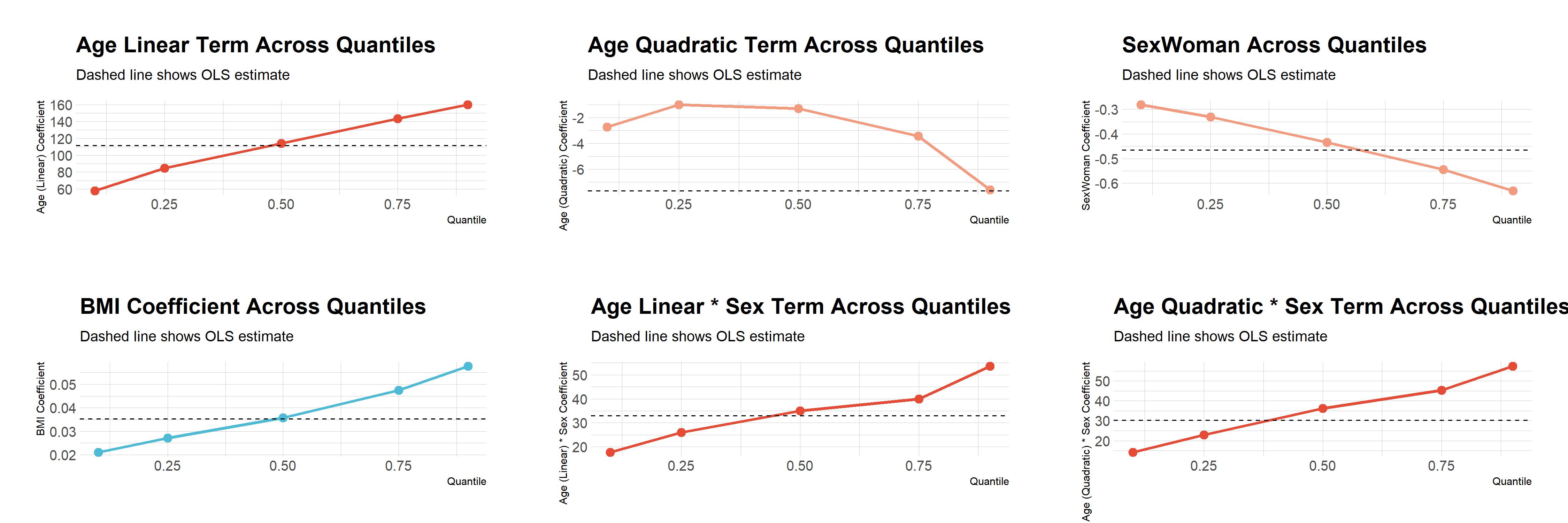

Supplement: Supplementary File 4 — Quantile regression coefficients for pulse wave velocity (PWV) across the distribution. This figure is the same plot as in Figure 4 except we used only 1/1000 of the data. [file Image4.jpeg]

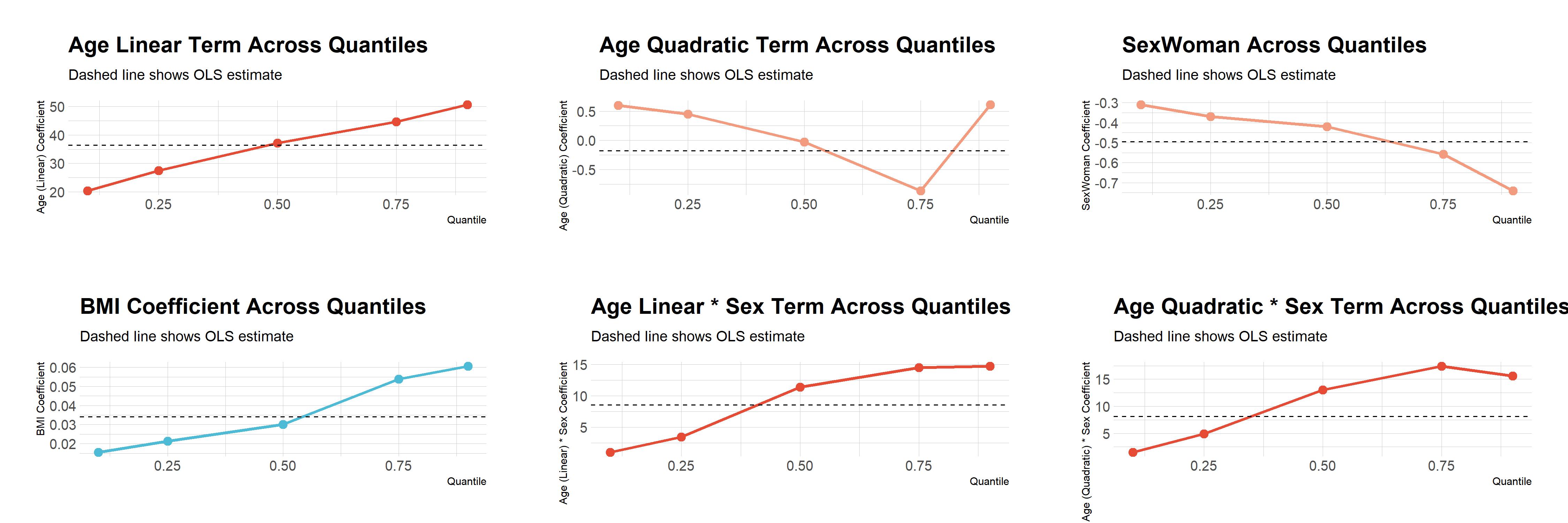

Supplement: Supplementary File 5 — Quantile Regression Coefficients for Pulse Wave Velocity (PWV) across the Distribution. This figure is the same plot as in Figure 4 except we used only 1/10000 of the data. [file Image5.jpeg]
